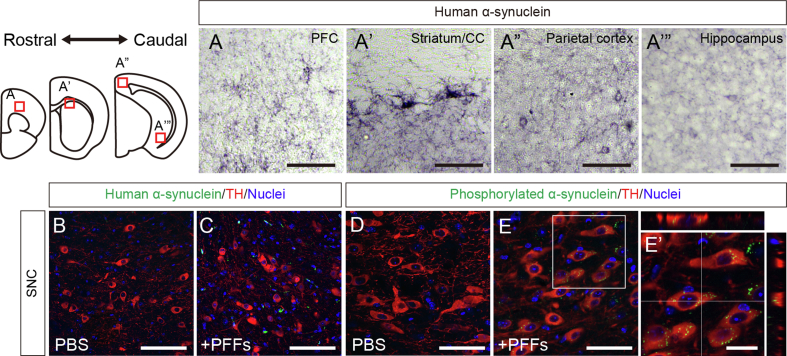# Host-to-graft propagation of inoculated α-synuclein into transplanted human induced pluripotent stem cell-derived midbrain dopaminergic neurons

**DOI:** 10.1016/j.reth.2023.12.019

**Published:** 2024-01-06

**Authors:** Serina Gima, Kazuya Oe, Kaneyasu Nishimura, Takashi Ohgita, Haruka Ito, Hiroyuki Kimura, Hiroyuki Saito, Kazuyuki Takata

**Affiliations:** a Joint Research Laboratory, Division of Integrated Pharmaceutical Science, Kyoto Pharmaceutical University, Kyoto 607-8414, Japan; b Laboratory of Functional Brain Circuit Construction, Graduate School of Brain Science, Doshisha University, Kyotanabe 610-0394, Japan; c Center for Instrumental Analysis, Kyoto Pharmaceutical University, Kyoto 607-8414, Japan; d Laboratory of Biophysical Chemistry, Kyoto Pharmaceutical University, Kyoto 607-8414, Japan; e Department of Analytical and Bioinorganic Chemistry, Kyoto Pharmaceutical University, Kyoto 607-8414, Japan; f Division of Probe Chemistry for Disease Analysis/Central Institute for Radioisotope Science, Research Center for Experimental Modeling of Human Disease, Kanazawa University, Kanazawa 920-8640, Japan

**Keywords:** Human induced pluripotent stem cells, Dopaminergic neurons, Cell transplantation, α-synuclein

## Abstract

**Introduction:**

Cell therapeutic clinical trials using fetal mesencephalic tissue provided a proof-of-concept for regenerative therapy in patients with Parkinson's disease. Postmortem studies of patients with fetal grafts revealed that α-synuclein^+^ Lewy body (LB)-like inclusions emerged in long-term transplantation and might worsen clinical outcomes even if the grafts survived and innervated in the recipients. Various studies aimed at addressing whether host-derived α-synuclein could be transferred to the grafted neurons to assess α-synuclein^+^ inclusion appearance in the grafts. However, determining whether α-synuclein in the grafted neurons has been propagated from the host is difficult due to the intrinsic α-synuclein expression.

**Methods:**

We induced midbrain dopaminergic (mDA) neurons from human induced pluripotent stem cells (hiPSCs) and transplanted them into the striatum of immunodeficient rats. The recombinant human α-synuclein preformed fibrils (PFFs) were inoculated into the cerebral cortex after transplantation of *SNCA*^−/−^ hiPSC-derived mDA neural progenitors into the striatum of immunodeficient rats to evaluate the host-to-graft propagation of human α-synuclein PFFs. Additionally, we examined the incorporation of human α-synuclein PFFs into *SNCA*^−/−^ hiPSC-derived mDA neurons using *in vitro* culture system.

**Results:**

We detected human α-synuclein-immunoreactivity in *SNCA*^−/−^ hiPSC-derived mDA neurons that lacked endogenous α-synuclein expression *in vitro*. Additionally, we observed host-to-graft α-synuclein propagation into the grafted *SNCA*^−/−^ hiPSC-derived mDA neurons.

**Conclusion:**

We have successfully proven that intracerebral inoculated α-synuclein PFFs are propagated and incorporated from the host into grafted *SNCA*^−/−^ hiPSC-derived mDA neurons. Our results contribute toward the basic understanding of the molecular mechanisms related to LB-like α-synuclein deposit formation in grafted mDA neurons.

## Highlights

• mDA neural progenitors were robustly induced from hiPSCs and gave rise to mDA neurons.
• hiPSC-derived mDA neurons survived in the striatum of immunodeficient rats.
• Human wild type α-synuclein PFFs were incorporated into *SNCA*^−/−^ hiPSC-derived mDA neurospheres *in vitro.*
• Human wild-type α-synuclein PFFs were propagated into *SNCA*^−/−^ hiPSC-derived mDA neurons in the grafts.

## 1 Introduction

Parkinson's disease (PD) is an age-related neurodegenerative disorder characterized by progressive loss of midbrain dopaminergic (mDA) neurons, leading to motor symptoms (e.g., resting tremor, rigidity, and bradykinesia) that are potentially treatable by symptomatic therapy in the early stage of disease. Pathological features from postmortem studies include accumulation of α-synuclein protein in Lewy bodies (LBs) that appear in the remaining mDA neurons of patients with PD [1]. Moreover, α-synuclein^+^ aggregates and neurites widely propagated across the brain in a prion-like manner corresponding to the neuropathological stage of PD [2].

Former cell transplantation studies in patients with PD provide a proof-of-concept for regenerative therapeutic strategy using a fetal mesencephalic tissue. In successful cases, grafted fetal mesencephalic tissue has survived, got innervated in the host brain, and improved the quality of life of the recipients with long-term symptomatic relief [3,4]. However, ethical issues and unstable fetal tissue supply make the standardized cell transplantation therapy difficult. Instead of fetal mesencephalic tissue, human embryonic stem cell (hESC) and human induced pluripotent stem cell (hiPSC)-derived mDA neurons have emerged as alternative sources of cell transplantation therapy. Recently, the stem cell technological advantages have allowed for scalable generation of highly purified hESC/hiPSC-derived mDA neurons, demonstrating survival of the long-term grafted mDA neurons and improvement of motor functions in nonhuman primate and rodent PD models [[5], [6], [7], [8]]. Furthermore, the standardized protocols provide hESC/hiPSC-derived mDA progenitors under good manufacturing practice conditions for clinical trials of cell transplantation therapy for patients with PD [9,10].

However, a drawback was observed in the postmortem studies of patients with PD who received fetal transplants, demonstrating LB-like α-synuclein deposit appearance in long-term grafts [[11], [12], [13], [14]]. Additionally, α-synuclein in the grafts could be transferred from the host into grafts in a prion-like manner. Furthermore, a study of a patient with PD having received unilateral fetal transplantation in the putamen revealed that grafted mDA neurons survived in the host brain and recovered motor functions in the first decade. However, this patient gradually lost motor functions from 14 years post-transplantation. A postmortem study revealed LB-like α-synuclein^+^ inclusions in 24-year grafts [15]. This clinical case suggested that even if grafted mDA neurons survived and got innervated in the host brain, their therapeutic effects might be lost due to synucleinopathy in the long-term follow-up. To reveal the underlying molecular mechanisms of α-synuclein^+^ inclusion in the grafted neurons, several studies aimed at assessing how host-derived α-synuclein is incorporated into the neuronal grafts derived from healthy rodent fetal mesencephalic tissue- and hESC-derived mDA neurons [[16], [17], [18]]. However, intrinsic α-synuclein expression in the grafted neurons might affect host-to-graft extrinsic α-synuclein transfer observations. Herein, we inoculated human α-synuclein preformed fibrils (PFFs) into the brain of rats with X-linked severe combined immunodeficiency (X-SCID) and demonstrated that α-synuclein PFFs got incorporated into *SNCA*^−/−^ hiPSC-derived mDA neuronal grafts. Since *SNCA*^−/−^ hiPSC-derived mDA neurons do not express endogenous α-synuclein protein, our results visibly indicated that host-derived human α-synuclein PFFs could be transmitted into grafted hiPSC-derived mDA neurons.

## 2 Materials and methods

### 2.1 Animals

We purchased 8-week-old wild-type C57BL/6 mice from Japan SLC Inc. (Hamamatsu, Japan). F344-*Il2rg*^*em1Iexas*^ X-SCID rats were supplied by the National BioResource Project - Rat, Kyoto University (Kyoto, Japan) [19]. The animals were kept at 25 °C under a 12-h light/dark cycle with *ad libitum* access to food and water in the Bioscience Research Center at Kyoto Pharmaceutical University. We performed the animal experiments in accordance with the National Institutes of Health Guide for the Care and Use of Laboratory Animals. Our protocols were approved by the Committee for Animal Research, Kyoto Pharmaceutical University.

### 2.2 hiPSC culture

The 1231A3 hiPSC line derived from ePBMC®, purchased from Cellular Technology Limited (http://www.immunospot.com/), it was established by Kyoto University, and provided by the RIKEN Bioresource Research Center (Tsukuba, Japan) through the National BioResource Project of the Ministry of Education, Culture, Sports, Science, and Technology/Japan Agency for Medical Research and Development (MEXT/AMED), Japan [20]. Herein, we used *SNCA*^−/−^ hiPSCs generated from the 1231A3 hiPSC line [21]. The hiPSC-related experiments were approved by the Ethical Review Committee for Medical and Health Research Involving Human Subjects, Kyoto Pharmaceutical University.

### 2.3 mDA neuronal induction from hiPSCs

mDA neuronal differentiation was performed as described previously with modifications [22,23]. Briefly, single-cell dissociated hiPSCs were plated on iMatrix511 silk-coated dishes at a density of 500,000 cells/cm^2^ in E6 medium (Thermo Fisher Scientific, Waltham, MA, USA) supplemented with 1 % nonessential amino acids (Fujifilm Wako Chemicals, Osaka, Japan), 1 % GlutaMax (Thermo Fisher Scientific), 0.1 mM 2-mercaptoethanol (Fujifilm Wako Chemicals), and 1 % penicillin/streptomycin (Fujifilm Wako Chemicals). For the first 24 h, 10 μM Y27632 (Selleck, Houston, TX, USA) was added in the medium. mDA neuronal induction was performed by supplementing the medium with LDN193189 (200 nM, Days 0–11, Selleck Chemicals), A83-01 (500 nM, Days 0–6, Fujifilm Wako Chemicals), purmorphamine (1 μM, Days 1–11 and 2 μM, Day 12–16, Selleck Chemicals), and CHIR99021 (1.5 μM, Days 3–11 and 7.5 μM, Days 12–16, Selleck Chemicals). The medium was gradually changed to Neurobasal medium (Thermo Fisher Scientific) with B-27 Supplement minus vitamin A (Thermo Fisher Scientific), 1 % GlutaMax, and 1 % penicillin/streptomycin between Days 5–11. On Day 16, the cells were dissociated into single cells, replated at a density of 750,000 cells/cm^2^ on iMatrix511-silk-coated dishes, and were cultured with Neurobasal medium with B-27 Supplement minus vitamin A, 1 % GlutaMax, and 1 % penicillin/streptomycin. mDA neuronal maturation was performed in the medium supplemented with GW3965 (10 μM, Days 16–21, Selleck Chemicals), DAPT (10 μM, Days 16–111, Selleck Chemicals), brain-derived neurotrophic factor (20 ng/mL, Days 16–111, Peprotech, Rocky Hill, NJ, USA), and ascorbic acid (200 μM, Days 16–111, Sigma-Aldrich, St. Louis, MO, USA), SU5402 (5 μM, Days 21–28, Selleck Chemicals), PD325901 (1 μM, Days 21–28, Selleck Chemicals). Finally, glial cell-derived neurotrophic factor (10 ng/mL, Days 21–111, Peprotech), dbcAMP (500 μM, Days 21–111, Selleck), and transforming growth factor β3 (1 ng/mL, Days 21–111, Peprotech) were added to the medium.

For the neurosphere culture, hiPSCs were dissociated into single cells and reseeded at a density of 9000 cells/well on ultra-low attachment V-bottom 96-well plates (Thermo Fisher Scientific) in the above-described differentiation medium and maintained until Day 60.

### 2.4 Recombinant human wild-type α-synuclein PFF preparation

Recombinant human α-synuclein PFFs was prepared as previously described [24]. Briefly, lyophilized α-synuclein was dissolved in 20 mM glycine buffer (pH 8.0) and solubilized by adding 2 M NaOH [25]. The solution was centrifuged at 10,000×*g* for 30 min at 4 °C to remove the insoluble aggregates after dialysis against PBS overnight. The supernatant containing monomeric α-synuclein (1 mg/mL) was incubated at 37 °C for 5 days with rotation for PFF production.

### 2.5 Thioflavin T (ThT) fluorescence assay

α-synuclein PFF formation was verified using an amyloid-specific fluorescent dye, ThT, which was added to a 10-times diluted α-synuclein solution up to a final concentration of 10 μM. The ThT fluorescent spectra were measured using a Hitachi F-2500 fluorescence spectrometer at 25 °C. The spectra were recorded between 450 and 600 nm at an excitation of 440 nm.

### 2.6 Far-ultraviolet (UV) circular dichroism (CD) spectroscopy

Far-UV CD spectra were recorded between 190 and 260 nm at 25 °C using a Jasco J-1500 spectropolarimeter (JASCO, Tokyo, Japan). The results were corrected by subtracting the buffer values.

### 2.7 Atomic force microscopy (AFM)

The α-synuclein PFF solution was deposited on freshly cleaved mica (The Nilaco Corporation, Tokyo, Japan) and incubated for 10 min. After washing the mica with distilled water, sample imaging was performed under ambient conditions at room temperature using a NanoScope IIIa Tapping Mode AFM (Veeco, Plainview, NY, USA) and microcantilever OMCLAC160TS-R3 (Olympus, Tokyo, Japan).

### 2.8 *SNCA*^−/−^ hiPSC-derived mDA sphere exposure to human α-synuclein PFFs

*SNCA*^−/−^ hiPSC-derived mDA neurospheres were exposed to α-synuclein PFFs (5 μg/mL) on Day 42. The medium was changed 2 days after exposure and the cells were fed every 2–3 days until Day 56.

### 2.9 Immunocytochemistry

We performed immunocytochemistry as previously described [26]. Briefly, we fixed the cells and neurospheres in 4 % paraformaldehyde (PFA) for 30 min at 4 °C. Next, we embedded the neurospheres in an optimal cutting temperature (OCT) compound (Sakura Finetek Japan Co., Ltd., Osaka, Japan) and performed sample sectioning at a thickness of 20 μm using a cryostat. We then blocked the fixed samples for 1 h at room temperature using a 5 % normal donkey serum in 0.1 M phosphate-buffered saline containing 0.1 % Triton-X (PBST), incubated at 4 °C overnight them with primary antibodies (Table 1), followed by an incubation with Alexa Fluor-labeled secondary antibodies (1:500; ThermoFisher) at room temperature for 2 h in the dark. Finally, we counterstained the cell nuclei using Hoechst 33342 (Dojindo, Kumamoto, Japan).

**Table 1:** List of antibodies.

Antigen	Species	Dilution	Catalog #	Vender
**Immunocytochemistry**				
DCX	Mouse	1:500	sc-271390	SantaCruz
FOXA2	Goat	1:500	AF2400	R&D Systems
Human α-synuclein	Mouse	1:500	sc-58480	SantaCruz
LMX1	Rabbit	1:3000	AB10533	Millipore
MAP2	Chicken	1:1000	ab5392	Abcam
OTX2	Goat	1:500	AF1979	R&D Systems
SOX2	Rabbit	1:500	AB5603	Millipore
Synapsin	Rabbit	1:500	57477	Millipore
TH	Rabbit	1:500	AB152	Millipore
TH	Mouse	1:500	MAB318	Millipore
**Immunohistochemistry**				
FOXA2	Goat	1:1000	AF2400	R&D Systems
hNuclei	Mouse	1:500	MAB1281	Millipore
Ki67	Mouse	1:100	NCL-L-Ki67-MM1	Leica
TH	Rabbit	1:1000	MAB318	Millipore
Human α-synuclein	Mouse	1:500	sc-58480	SantaCruz
α-synuclein (phospho S129)	Mouse	1:500	015-25191	WAKO
**DAB staining**				
hNCAM	Mouse	1:5000	sc-106	SantaCruz
Human α-synuclein	Mouse	1:5000	sc-58480	SantaCruz

### 2.10 Cell transplantation

We used either male or female F344-*Il2rg*^*em1Iexas*^ X-SCID rats in this study. We dissociated hiPSC-derived mDA progenitors into single cells for cell transplantation on Day 16 using Accutase (Nacalai Tesque, Kyoto, Japan) at 37 °C for 10 min, then prepared a cell suspension of approximately 1 × 10^5^ cells/μL in 10 μM Y27632-supplemented PBS(−). We anesthetized the animals through intraperitoneal injection with a mixture of medetomidine (0.3 mg/kg, ZENOAQ, Koriyama, Japan), midazolam (4 mg/kg, Maruishi Pharmaceutical Co., Osaka, Japan), and butorphanol (5 mg/kg, Meiji Seika Pharma, Tokyo, Japan). Finally, we performed cell transplantation through stereotactic injection of 4 × 10^5^ cells in a volume of 4 μL (1 μL/min) through a 22-gauge needle into the right striatum (from the bregma: A +0.5; L +3.0, V +3.0 and +4.0, and TB 0).

### 2.11 α-synuclein PFF inoculation into the animal brain

The animals were anesthetized by an intraperitoneal injection of a mixture comprising medetomidine (0.3 mg/kg), midazolam (4 mg/kg), and butorphanol (5 mg/kg). To mice, α-synuclein PFFs were inoculated with a stereotactic injection of 5 μg in 2 μL (1 μL/min) through a 26-gauge needle into the right striatum (from the bregma: A +0.5; L +2.0; V +3.0 and + 4.0; TB 0). To X-SCID rats, α-synuclein PFFs were inoculated with a stereotactic injection of 4 μg in a volume of 2 μL (1 μL/min) per site through a 26-gauge needle into the right cerebral cortex (from the bregma: A +2.0; L +4.0; V +2.0; TB 0 and A +1.0; L +1.0, V +2.0; TB 0).

### 2.12 Immunohistochemistry

We performed the DAB staining and immunofluorescence as described previously [27]. Briefly, we post-fixed the brain samples in 4 % PFA for 2 days and then transferred the samples into a 30 % sucrose solution at 4 °C. Subsequently, we embedded the brain samples in the OCT compound and cut them to 40-μm-think sections.

### 2.13 Histological analysis evaluation

We determined the graft volume through hNCAM-positive area identification in every sixth section using a BZ-X800 microscope (Keyence, Osaka, Japan) and totaling the volumes of whole tall cylinders according to Cavalieri's principle. We estimated the immunoreactive cell numbers in each graft by manual cell counting in every sixth section.

### 2.14 5-ethynyl-2′-deoxyuridine (EdU) labeling *in vivo*

We performed the EdU labeling using Click-iT EdU Cell Proliferation Kit for Imaging, Alexa Fluor 488 dye (Thermo Fisher Scientific). X-SCID rats were administrated with EdU (50 mg/kg) through intraperitoneal injection 4 h before sacrifice. We performed signal detection according to the manufacturer's protocol.

### 2.15 Statistical analysis

The values are represented as the mean ± standard deviation or standard error of the mean. The means of the two groups were compared using independent samples Student's *t*-test. All statistical analyses were conducted using Prism 10 (GraphPad, San Diego, CA, USA).

## 3 Results

### 3.1 Robust hiPSC-derived mDA neuronal induction

**Fig. 1:**
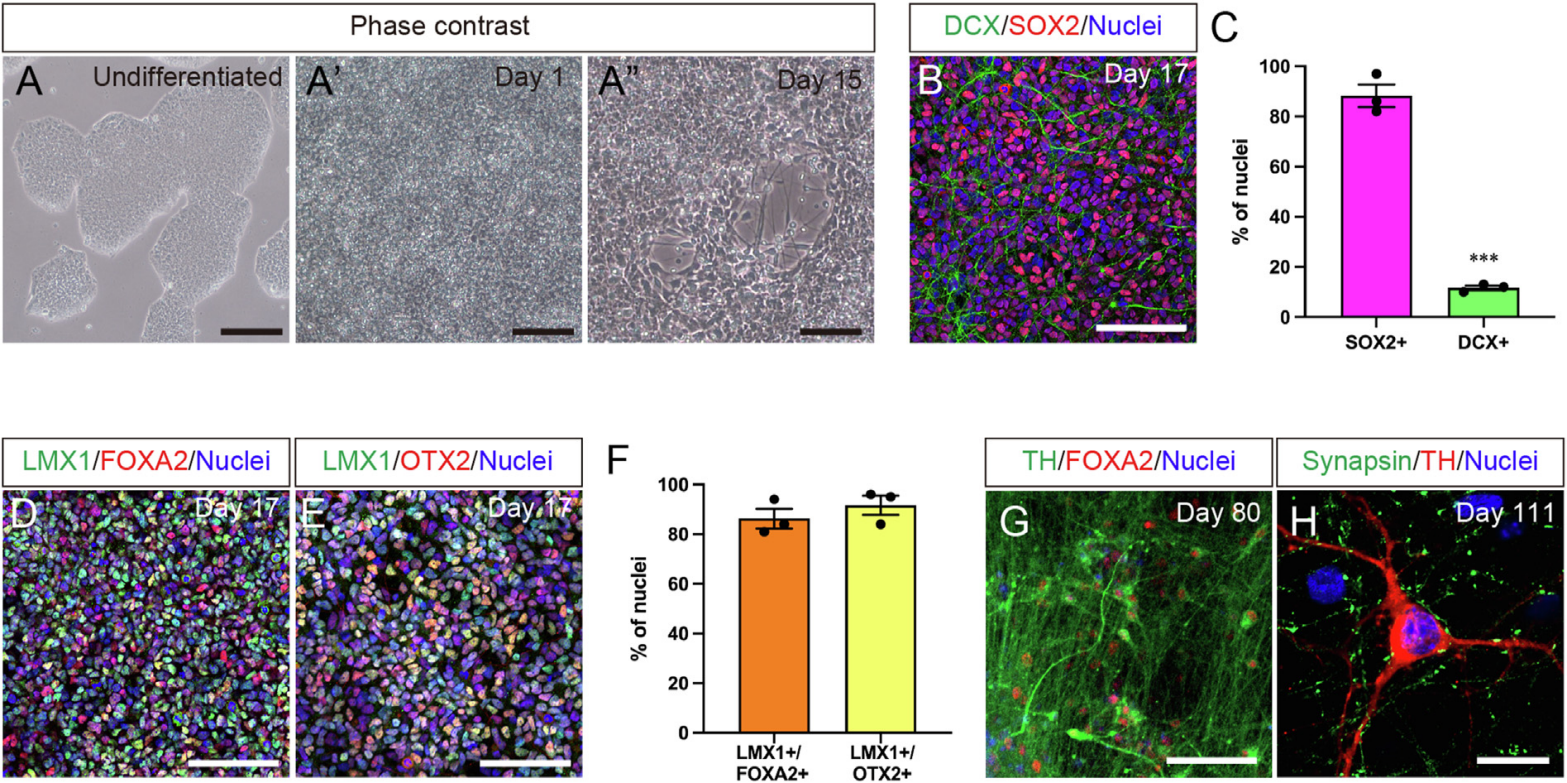
hiPSC-derived mDA neuronal induction. Phase contrast images of undifferentiated hiPSCs (**A**) and differentiating cells on Days 1 (**A′**) and 15 (**A″**). Scale bars: (**A**) 200 μm and (**A′** and **A″**) 100 μm. (**B**) SOX2 and DCX immunostaining on Day 17. Scale bar: 100 μm. (**C**) SOX2^+^ and DCX^+^ cell proportions on Day 17. Significance by Student's t-test: ∗∗∗*p* < 0.001. (**D and E**) LMX1 and FOXA2 immunostainings on Day 17. Scale bar: 100 μm. (**F**) LMX1^+^ and FOXA2^+^ cell proportions on Day 17. (**G**) TH and FOXA2 immunostaining on Day 80 and (**H**) TH and synapsin immunostaining on Day 111. Scale bars: (**G**) 100 μm and (**H**) 20 μm.

We generated mDA neuronal progenitors from hiPSCs according to a previous report with modifications [22,23]. Minimal compound combinations for mDA neuronal progenitor induction were supplemented during hiPSC differentiation in a chemically defined E6 medium under a monolayer feeder-free culture system. Briefly, the neuronal fate and ventral midbrain identity were induced by SMAD inhibitors LDN193189 and A83-01 and the combination of the Smoothened receptor agonist purmorphamine and GSK3β inhibitor CHIR99021, respectively. The cell morphology drastically changed during neuronal differentiation, and the cells exhibited neuronal progenitor morphology with neurites at Day 15 (Fig. 1A, A', and A”). hiPSCs were differentiated into SOX2-expressing neuronal progenitors (SOX2^+^/nuclei: 88.3 % ± 4.5 %, n = 3), whereas the proportion of DCX^+^ immature neurons per total cells were 11.7 % ± 0.9 % (n = 3) on Day 17 (Fig. 1B and C). In addition, hiPSC-derived neuronal progenitors acquired ventral midbrain identity, defined by LMX1, FOXA2, and OTX2 expression with high proportions per total cells (LMX1^+^ and FOXA2^+^/nuclei: 86.3 % ± 3.9 % and LMX1^+^ and OTX2^+^/nuclei: 91.7 % ± 3.8 %, n = 3) (Fig. 1D–F). For long-term maturation cultures, hiPSC-derived neuronal progenitors gave rise to TH^+^ mDA neurons co-expressing FOXA2 on Day 80 (Fig. 1G). Extensive mDA maturation by Day 111 revealed that TH^+^ neurons expressed synapsin, a pre-synaptic marker (Fig. 1H). These results indicated robust hiPSC-derived mDA neuronal progenitor induction, giving rise to mDA neurons.

### 3.2 Histological verification of grafted hiPSC-derived cells

**Fig. 2:**
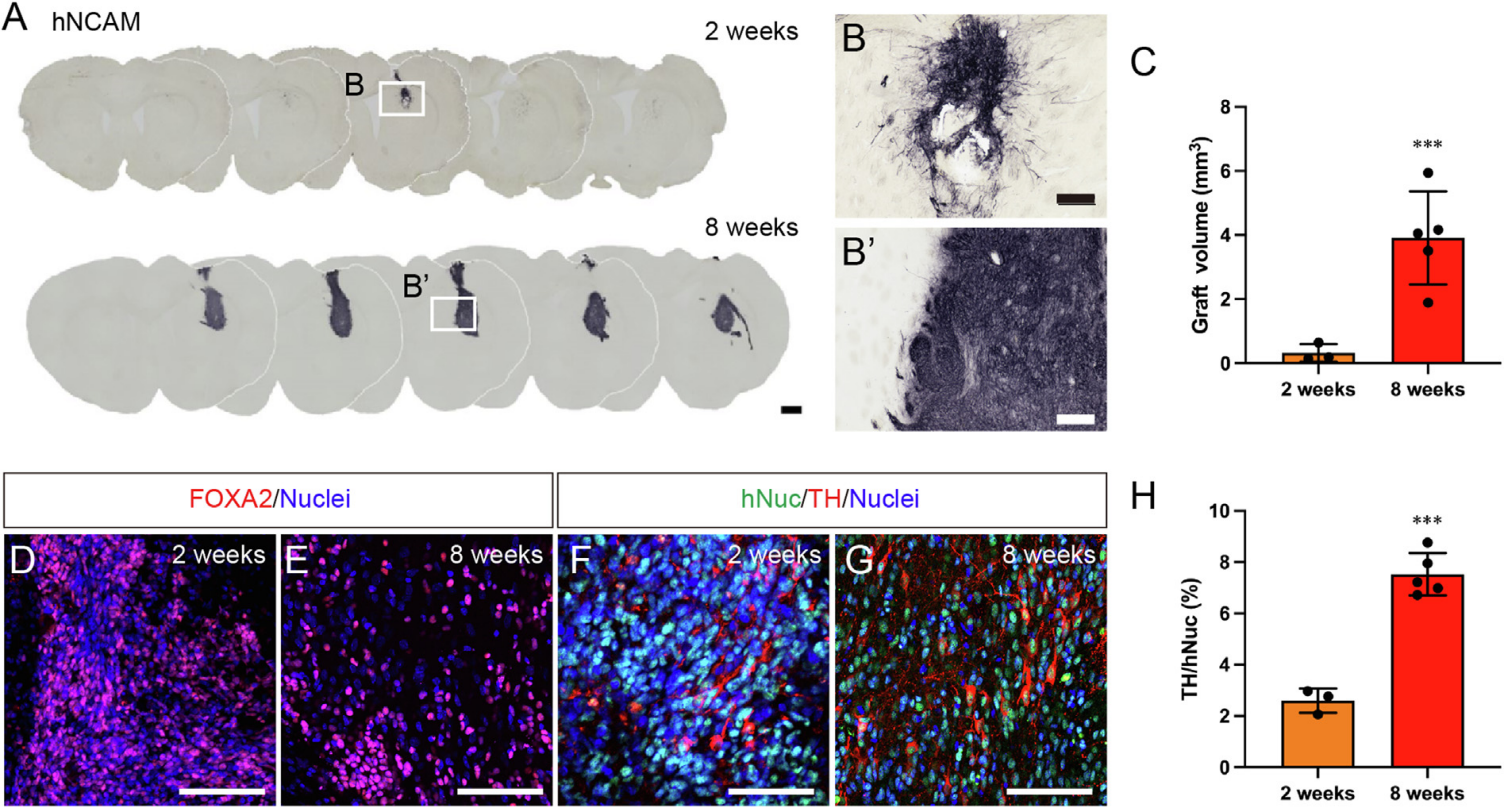
Histological analysis of grafted hiPSC-derived mDA neurons. (**A, B, and B′**) hNCAM immunostaining on weeks 2 and 8 post-transplantation. Scale bars: (**A**) 1 mm and (**B and B′**) 200 μm. (**C**) hiPSC-derived neuron graft volume quantification. Significance by Student's t-test: ∗∗∗*p* < 0.001. (**D** and **E**) FOXA2 and (**F** and **G**) hNuc and TH immunostaining on weeks 2 and 8 post-transplantation. Scale bar: 100 μm. (**H**) TH^+^ and hNuc^+^ cell proportions in the graft. Significance by Student's t-test: ∗∗∗*p* < 0.001.

**Figure S1:**
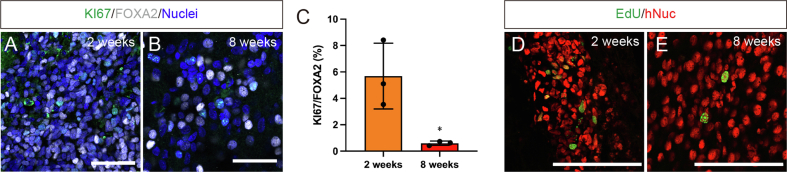


Next, we transplanted the hiPSC-derived mDA neuronal progenitors after 16 days of differentiation into the striatum of X-SCID rats. The X-SCID rats were already validated for human cell-derived graft xeno-transplantation into the brain [19,28]. Our immunohistochemical analysis using an anti-human neuronal cell adhesion molecule (hNCAM) antibody indicated that human neuronal cells survived in the striatum at weeks 2 and 8 post-transplantation (Fig. 2A, B, and B’), and the graft volume at week 8 significantly increased compared to that at week 2 (0.32 ± 0.28 mm^3^ and 3.91 ± 1.28 mm^3^ at weeks 2 and 8, respectively, n = 3–5) (Fig. 2C). FOXA2^+^ cells could be dominantly observed in the grafts at both weeks 2 and 8 post-transplantation (Fig. 2D and E). The proliferative ability of the grafted cells was assessed by Ki67^+^ cells and EdU incorporation. The Ki67^+^ and FOXA2^+^ cell ratio on week 8 significantly decreased compared to that on week 2 post-transplantation (5.68 % ± 1.44 % and 0.60 % ± 0.09 % at weeks 2 and 8, respectively, n = 3) (Supplementary Fig. S1A–C). Correspondingly, EdU^+^ cell numbers also reduced by week 8 compared to those at week 2 post-transplantation (4.56 % ± 0.77 % and 0.54 % ± 0.14 % at weeks 2 and 8, respectively, n = 2–3) (Supplementary Fig. S1D and E). In addition, human nuclear protein (hNuc)^+^ and TH^+^ neurons were detected both 2 and 8 weeks after transplantation. The TH^+^ neuron ratio at week 8 increased compared to that at week 2 (2.60 % ± 0.48 % and 7.53 % ± 0.65 % at weeks 2 and 8, respectively, n = 3–5) (Fig. 2F–H). These results indicated that grafted hiPSC-derived mDA progenitors exited the cell cycle and gave rise to TH^+^ mDA neurons in the X-SCID rat brain.

### 3.3 α-synuclein PFF incorporation into *SNCA*^−/−^ hiPSC-derived mDA neurospheres

**Fig. 3:**
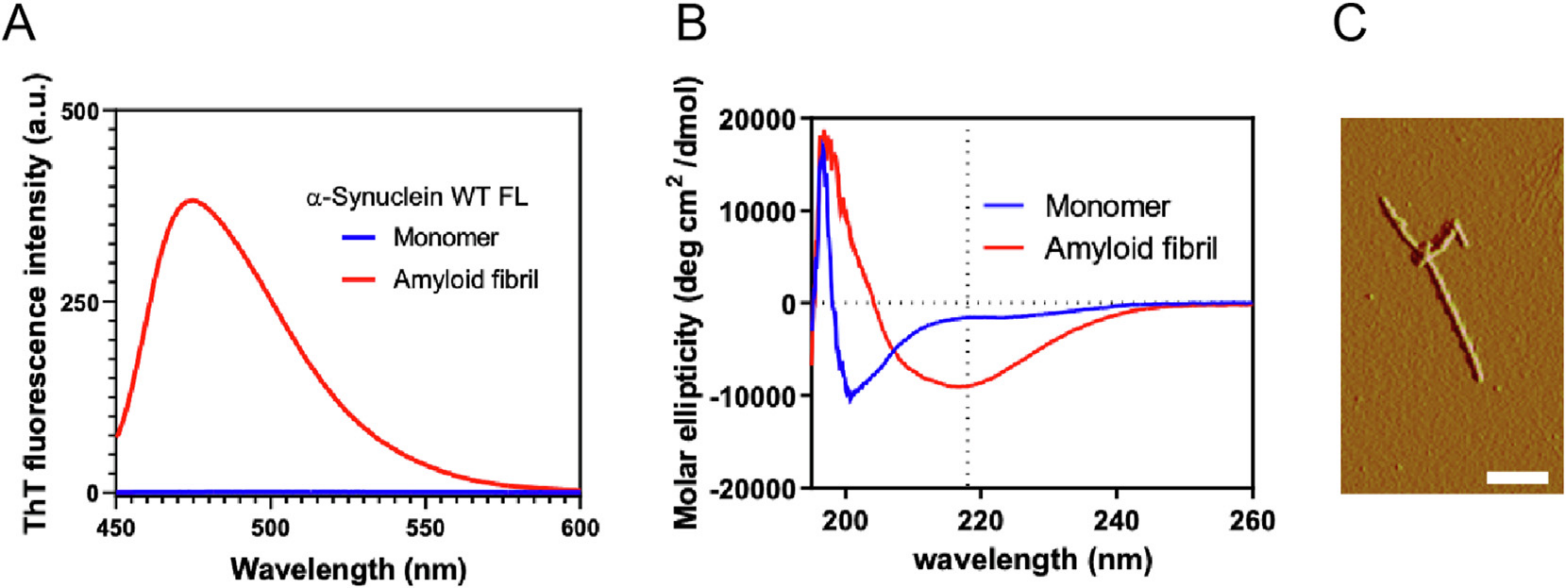
Human α-synuclein PFF characterization. (**A**) ThT fluorescence emission spectra excited at 440 nm for α-synuclein before (blue) and after (red) incubation at 37 °C for five days. (**B**) FarUV CD spectra before (blue) and after (red) incubation at 37 °C for five days. (**C**) AFM image of α-synuclein fibrils. Scale bar: 500 nm.

To investigate α-synuclein transmission into the hiPSC-derived mDA neurons, we first synthesized recombinant human α-synuclein PFFs under a cell-free system [24]. After the 5-day incubation of recombinant human α-synuclein with rotation, we observed an increased fluorescence intensity of the amyloid-specific dye, ThT (Fig. 3A), and the CD spectra of α-synuclein changed from a single negative peak below 200 nm to a negative peak at approximately 220 nm (Fig. 3B), suggesting the random-coil-to-β-sheet-rich amyloid structure formation upon incubation. Furthermore, we confirmed thin and straight fibril formation by the AFM imaging of the α-synuclein solution (Fig. 3C).

**Fig. 4:**
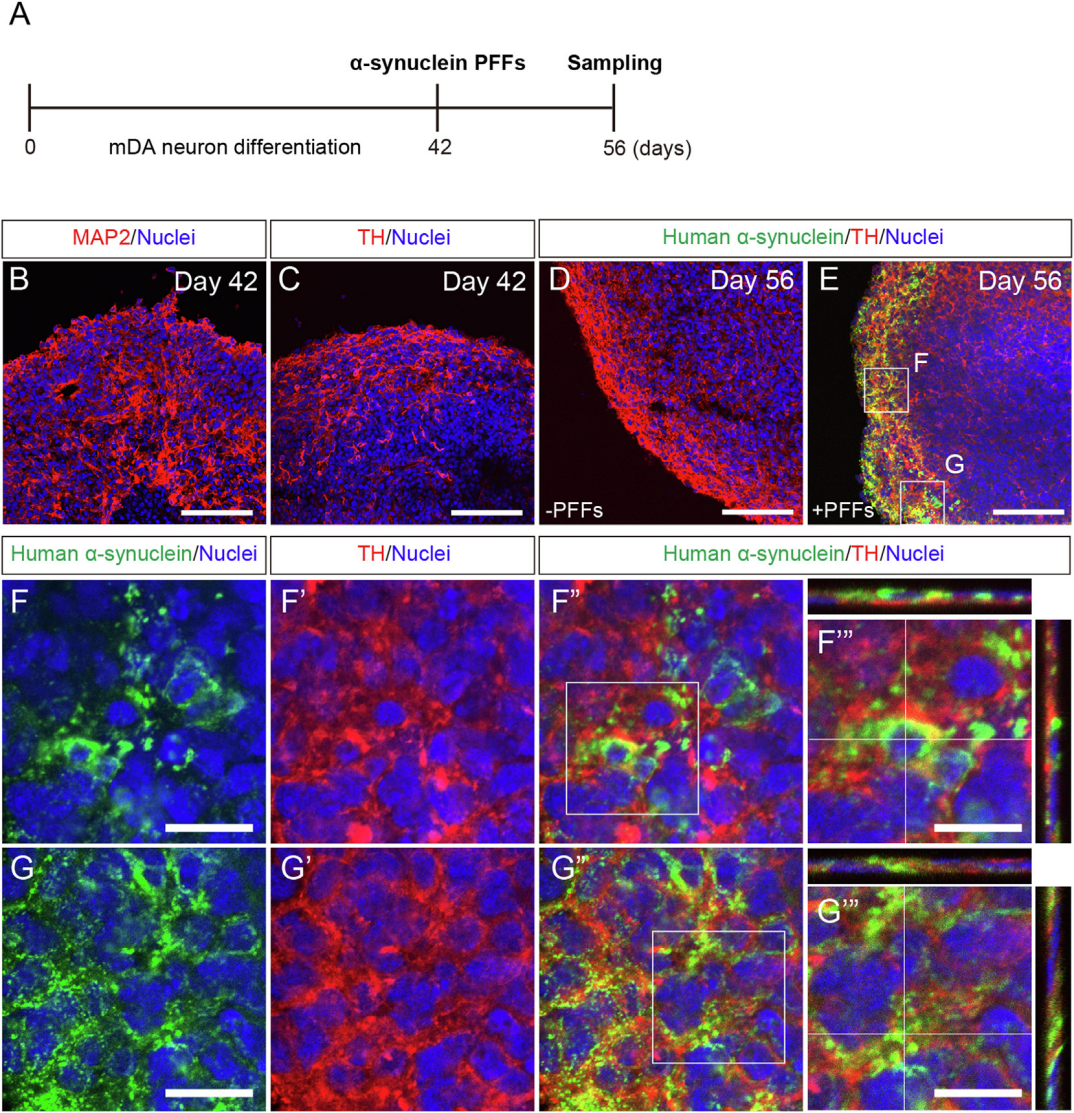
α-synuclein PFF incorporation into hiPSC-derived mDA neurons *in vitro.* (**A**) Experimental time course. (**B** and **C**) MAP2 and TH immunostaining in *SNCA*^*−/−*^ hiPSC-derived mDA neurospheres on Day 42. Scale bar: 100 μm. (**D** and **E**) Human α-synuclein and TH immunostaining in *SNCA*^*−/−*^ hiPSC-derived mDA neurospheres with or without α-synuclein PFF treatment on Day 56. Scale bar: 100 μm. (**F–F‴** and **G–G‴**) Representative image of cell body (**F–F‴**) and neurites (**G–G‴**) of human α-synuclein-incorporated TH^+^ cells. Scale bars: (**F** and **G**) 20 μm and (**F‴** and **G‴**) 10 μm.

Next, α-synuclein PFFs were exposed to *SNCA*^−/−^ hiPSC-derived mDA neurospheres to verify intracellular α-synuclein PFF incorporation. *SNCA*^−/−^ hiPSCs were generated using the CRISPR-Cas9 technology, never expressing endogenous α-synuclein protein after differentiation into mDA neurons [21]. Moreover, we verified that the mDA neuronal differentiation efficiency was not affected by the *SNCA* null mutation. We generated mDA neurospheres from *SNCA*^−/−^ hiPSCs as described previously [21] and exposed them to α-synuclein PFFs on Day 42 when MAP2^+^ and TH^+^ neurons emerged (Fig. 4A–C). Fourteen days after the α-synuclein PFF exposure, we observed human α-synuclein in the *SNCA*^−/−^ hiPSC-derived TH^+^ neurons on Day 56 (Fig. 4D and E). Since *SNCA*^−/−^ hiPSC-derived TH^+^ neurons lack the intrinsic α-synuclein protein expression, the immunoreactive α-synuclein signals should belong to the incorporated α-synuclein PFFs. Furthermore, our Z-stack imaging allowed us to detect human α-synuclein PFFs in TH^+^ cell bodies (Fig. 4F-F'") and their neurites (Fig. 4G-G'").

### 3.4 Inoculated α-synuclein propagation into grafted hiPSC-derived mDA neurons

To induce α-synuclein PFF propagation in the brain by resembling an established model, we inoculated α-synuclein PFFs in the striatum of healthy mice [29]. Sixteen weeks after the inoculation, human α-synuclein PFFs were widely distributed in the mouse brain, e.g., in the prefrontal cortex (PFC), striatum/corpus callosum (CC), parietal cortex, and hippocampus, far from the injected site (Supplementary Fig. S2A–A’”). In addition, we also detected human α-synuclein-immunoreactivity in the substantia nigra pars compacta (SNC) but not in that of noninoculated animals (Supplementary Fig. S2B and C). In addition, we detected phosphorylated α-synuclein in the TH^+^ neurons in the SNC of the inoculated mice but not in that of noninoculated animals (Supplementary Fig. S2D, E, and E’).

**Fig. 5:**
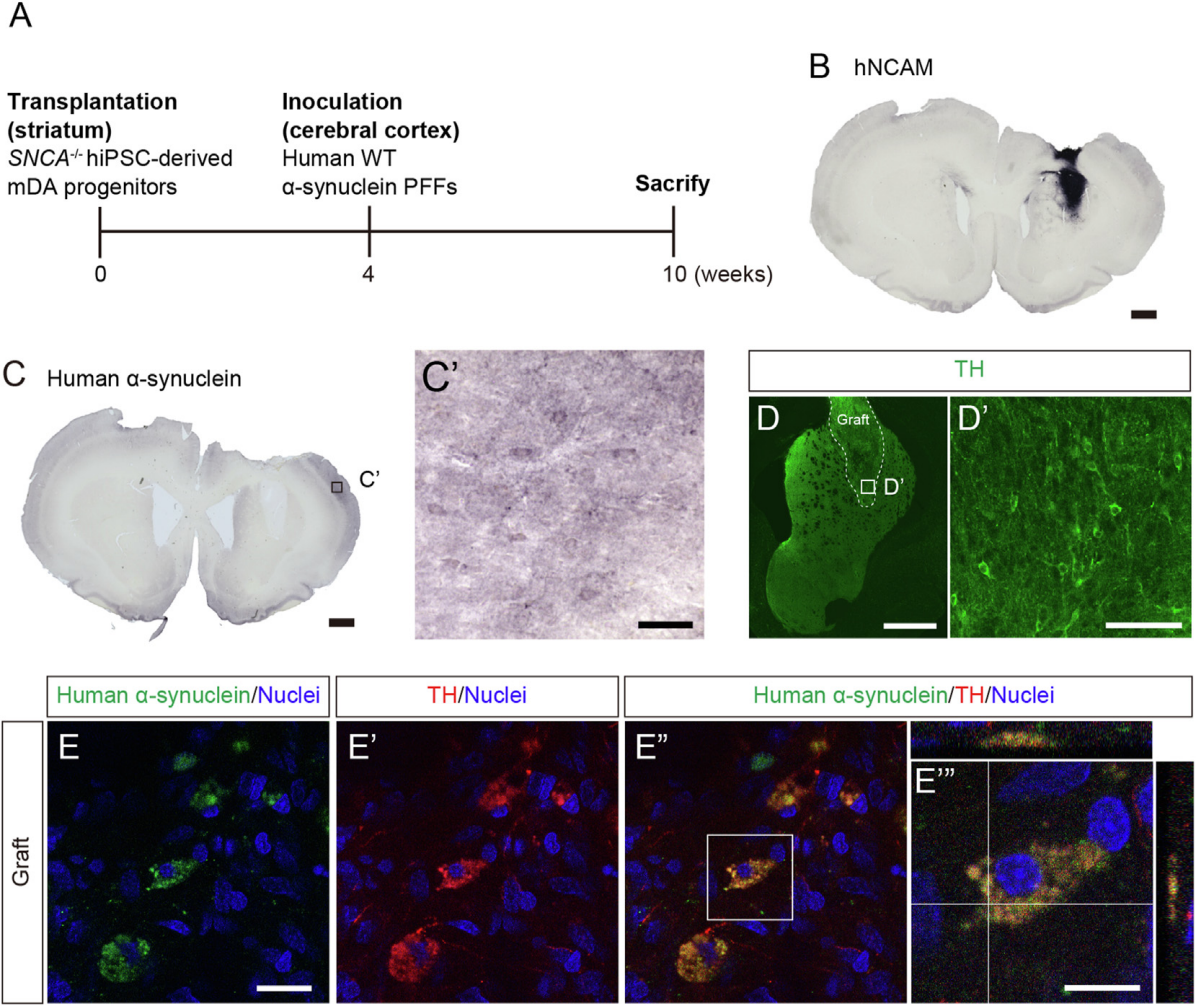
Histological analysis of inoculated α-synuclein PFF propagation into the *SNCA*^−/−^ hiPSC-derived mDA neuronal grafts. (**A**) Experimental time course. (**B**) hNCAM, (**C** and **C′**) human α-synuclein, and (**D** and **D′**) TH immunostaining on week 10 post-transplantation. Scale bars: (**B**, **C** and **D**) 1 mm and (**C′** and **D′**) 100 μm. (**E-E″**) Human α-synuclein and TH immunostaining in the grafts on week 10 post-transplantation. Scale bars: (**E**) 20 μm and (**E‴**) 10 μm.

Finally, we investigated whether inoculated α-synuclein PFFs would be propagated into grafted hiPSC-derived mDA neurons. In this experiment, we first transplanted *SNCA*^−/−^ hiPSC-derived mDA neuronal progenitors into the striatum of X-SCID rats. Four weeks after transplantation, α-synuclein PFFs were inoculated into the ipsilateral cerebral cortex, and the animals were subsequently sacrificed six weeks after inoculation (Fig. 5A). During our histological analysis, we observed hNCAM^+^ grafts in the striatum 10 weeks after transplantation (Fig. 5B). In addition, the inoculated human α-synuclein protein was spread from the injected site 6 weeks after inoculation (Fig. 5C and C′). *SNCA*^−/−^ hiPSC-derived mDA neurons also survived in the graft (Fig. 5D and D′). We detected human α-synuclein-immunoreactivity in the grafted *SNCA*^−/−^ hiPSC-derived TH^+^ neurons (Fig. 5E–E″). Our Z-stack images revealed that α-synuclein PFFs were incorporated into the grafted *SNCA*^−/−^ hiPSC-derived TH^+^ neurons (Fig. 5E’”). These results indicated that inoculated human α-synuclein PFFs were transmitted and incorporated into grafted *SNCA*^−/−^ hiPSC-derived mDA neurons.

## 4 Discussion

In this study, we managed to provide experimental evidence that grafted hiPSC-derived mDA neurons incorporated α-synuclein protein transmitted from the host brain. X-SCID rats were intracerebrally inoculated with human α-synuclein PFFs after the intrastriatal transplantation of the *SNCA*^*−/−*^ hiPSC-derived cells that never expressed endogenous α-synuclein. We used an anti-human α-synuclein antibody that specifically recognizes human α-synuclein and does not bind with rodent α-synuclein [30]. These experimental tools enabled us to successfully detect inoculated human α-synuclein PFF propagation into the grafted *SNCA*^−/−^ hiPSC-derived DA neurons and exclude the detection of intrinsic α-synuclein expression of human iPSC-derived neurons and the rodent brain.

Inclusion of α-synuclein, a main LB component is a neuropathological feature of PD [1]. Familial PD is reportedly coupled with increased *SNCA* gene copy numbers and missense mutations involved in DA neuronal cell death in the SNC, which is another pathological feature of PD [[31], [32], [33], [34], [35]]. Additionally, postmortem studies of patients with PD revealed that the aggregates of α-synuclein extended through the brain via interconnected neuronal pathways according to clinical symptom staging [2]. Moreover, synucleinopathy could be modeled by recombinant α-synuclein PFF inoculation into the animal brain and exposing it to cultured neurons [29,[36], [37], [38]]. In fact, we observed that intrastriatal inoculation of human α-synuclein PFFs led to their propagation through the brain and induced α-synuclein phosphorylation in healthy mouse SNC TH^+^ neurons.

Postmortem studies of the patients with PD who received fetal mesencephalic transplantation revealed LB acquisition in long-term grafts, potentially contributing to worsened clinical outcomes [[11], [12], [13], [14], [15]]. Therefore, several experimental approaches aimed at revealing the molecular mechanisms of synucleinopathy in the grafts, suggesting host-to-graft propagation [[16], [17], [18]]. Without a doubt, this study demonstrated that inoculated human α-synuclein PFFs propagated in the brain and were incorporated into the grafted *SNCA*^−/−^ hiPSC-derived mDA neurons. Since *SNCA*^−/−^ hiPSC-derived mDA neurons in the grafts never expressed intrinsic α-synuclein, the human α-synuclein immunoreactivity in the grafted cells could only be derived from the host brain.

A former study described that exogenous α-synuclein PFF seeding recruits unfolded endogenous α-synuclein to initiate the pathological aggregate formation and induces α-synuclein phosphorylation in the cells [37]. No such α-synuclein-related pathological changes occurred in the case of incorporation into *SNCA*^−/−^ cells, which were not expressing endogenous α-synuclein unlike their wild-type counterparts [29,38]. In coherence with previous reports, we did not detect any phosphorylated α-synuclein in *SNCA*^−/−^ hiPSC-derived mDA neurons either *in vivo* or *in vitro* (data not shown), although we observed inoculated α-synuclein incorporation in the grafted *SNCA*^−/−^ hiPSC-derived mDA neurons. To unravel the pathological changes of α-synuclein following the incorporation of the inoculated α-synuclein PFFs into the grafted hiPSC-derived mDA neurons, the grafts of *SNCA*^+/+^ hiPSC-derived mDA neurons should be conducted, since pathological changes of endogenous α-synuclein are induced by exogenous α-synuclein PFFs after incorporation into the cells. Indeed, various studies revealed that α-synuclein pathology in the grafted cells appear after the incorporation of exogenous α-synuclein [[16], [17], [18]].

Preclinical studies using positron emission tomography (PET) provided noninvasive imaging of dopamine reuptake in grafted hiPSC-derived DA neurons, dividing cell proliferation, and immune cell inflammation response to monitor the cellular aspects in long-term follow-up [5,8,39]. Recently, several highly potent PET tracers were developed to monitor synucleinopathy [[40], [41], [42], [43], [44]]. Since α-synuclein propagation and aggregation in the grafts might diminish cell transplantation therapeutic efficacy, pathological feature monitoring in the grafts displaying synucleinopathy might provide beneficial follow-up to support long-term symptomatic relief from the grafted neurons for better cell transplantation therapeutic outcomes. Moreover, several α-synuclein oligomer-binding compounds have recently emerged as disease-modifying therapeutic candidates to reduce synucleinopathy [[45], [46], [47], [48], [49], [50]], potentially applicable for improvement of α-synuclein propagation into the grafted hiPSC-derived mDA neurons to maintain the clinical outcomes as a disease-modified therapy in long-term follow-up.

In conclusion, we successfully proved that intracerebral inoculated α-synuclein PFFs are propagated and incorporated from the host into grafted *SNCA*^−/−^ hiPSC-derived mDA neurons that lacked endogenous α-synuclein expression. Our results contribute to the common understanding of the underlying molecular mechanisms related to LB-like α-synuclein deposit formation in grafted mDA neurons and the symptomatic therapeutic strategy targeting α-synuclein.

## Author contributions

S.G., K.O., and H.I. performed experiments. K.N. designed the project, wrote the manuscript, and made final approval of the manuscript. T.O. and H.S. provided recombinant human α-synuclein PFFs. H.K. provided technical support and helped to design the project. K.T. supervised the project.

## Declaration of competing interest

The authors declare that they have no known competing financial interests or personal relationships that could have appeared to influence the work reported in this paper.

## Figures and Tables

**Figure S2:**