# Supplementary material for: Host-to-graft propagation of inoculated α-synuclein into transplanted human induced pluripotent stem cell-derived midbrain dopaminergic neurons
Source: Regen Ther. 2024 Jan 6;25:229–37. doi: 10.1016/j.reth.2023.12.019 (PMC10818157; doi:10.1016/j.reth.2023.12.019)
Supplement: Multimedia component 1 [file mmc1.docx]

**Supplementary Figure legends**

**Supplementary Fig. S1. Histological analysis of dividing cells in the grafts.** (**A and** **B**) Ki67 and FOXA2 immunostaining on weeks 2 and 8 post-transplantation. Scale bar: 50 µm. (**C**) Ki67^+^ and FOXA2^+^ cell proportions in the graft. Significance by Student’s t-test: **p* < 0.05. (**D**, **E**) EdU and hNuc immunostaining on weeks 2 and 8 post-transplantation. Scale bar: 100 µm.

**Supplementary Fig. S2. Histological analysis of inoculated α-synuclein PFF propagation in the mouse brain.** (**A–A’”**) Human α-synuclein immunostaining in the mouse brain on week 16 post-inoculation of α-synuclein PFFs. Scale bar: 100 µm. (**B and C**) Human α-synuclein and TH in the SNC on week 16 post-inoculation of α-synuclein PFFs. Scale bar: 100 µm. (**D, E, and E’**) Phosphorylated α-synuclein and TH immunostaining in the SNC on week 16 post-inoculation of α-synuclein PFFs. Scale bars: (**D and E**) 50 µm and (**E’**) 20 µm. Abbreviations: CC, corpus callosum; PFC, prefrontal cortex; SNC, substantia nigra pars compacta.
